# Soil fungal community structure and ecological functions in fairy rings of *Leucocalocybe mongolica* in Bayanbulak grassland

**DOI:** 10.3389/fmicb.2025.1667514

**Published:** 2025-10-14

**Authors:** Senyuan Wang, Xingzhe Wang, Jiajia Xu, Rimai Na, Yanan Wang, Jie Wei, Fahu Li, Qiuli Bao

**Affiliations:** ^1^College of Life Sciences, Inner Mongolia University, Hohhot, China; ^2^Key Laboratory of Herbage and Endemic Crop Biology, Ministry of Education, Inner Mongolia University, Hohhot, China; ^3^College of Forestry, Inner Mongolia Agricultural University, Hohhot, China; ^4^Inner Mongolia Engineering Technology Research Center of Germplasm Resources Conservation and Utilization, Inner Mongolia University, Hohhot, China; ^5^Vocational and Technical College, Inner Mongolia Agricultural University, Baotou, China

**Keywords:** *Leucocalocybe mongolica*, fairy ring, soil fungal community, Bayanbulak grassland, microbial interaction network

## Abstract

*Leucocalocybe mongolica* is an ecologically important grassland fungus that forms distinct fairy rings, which are hotspots of soil microbial activity. In this study, we investigated soil fungal communities across three spatial zones of *L. mongolica* fairy rings (inside, fruiting, and outside zones) in the Bayanbulak alpine grassland of Xinjiang, China. High-throughput sequencing, co-occurrence network analysis, and functional prediction tools (FUNGuild and PICRUSt2) were applied to assess community structure, interactions, and environmental drivers. The fruiting zone was dominated by *L. mongolica*, showing reduced fungal diversity, simplified microbial networks, and increased laccase activity, whereas the outside zone maintained higher diversity and more complex interactions. Redundancy analysis revealed strong correlations between fungal community composition and nitrogen availability, laccase activity, and pH. Functional predictions indicated spatially distinct trophic strategies, with saprotrophs enriched in the fruiting zone and ectomycorrhizal fungi dominant in other zones. These findings suggest that elevated laccase activity and nitrogen cycling may be key processes through which *L. mongolica* influences soil fungal zonation in grassland ecosystems.

## 1 Introduction

*Leucocalocybe mongolica*, an endemic and representative species in Chinese grasslands, is widely recognized as a valuable germplasm resource due to its distinctive aroma, large basidiocarps, and fleshy texture (Lu et al., 2017; Zaki et al., 2022). It is rich in bioactive compounds (e.g., peptides and polysaccharides) and essential nutrients, and studies have confirmed its diverse medicinal properties, including hepatoprotective, anticancer, antioxidant, and immunomodulatory effects (Duan et al., 2021; Wang et al., 2019; Ma et al., 2022; Zhao et al., 2020; You et al., 2014; Zaki et al., 2024). In natural grassland ecosystems, *L. mongolica* often forms type II fairy rings (Shantz and Piemeisel, 1917; Fidanza, 2007; Fidanza et al., 2016; Ito et al., 2017), which are characterized by concentric, dark green bands of grass with dense, belt-like distributions of fruiting bodies, clearly distinguishable from the surrounding vegetation. Unlike type I rings, which typically cause turf necrosis, or type III rings, which promote luxuriant plant growth without distinct fruiting belts, type II rings represent the most visually recognizable form in natural grasslands (Caesar-Tonthat et al., 2013). Fairy rings play a crucial ecological role by forming complex interactions among fungi, soil microbes, and plant roots, which may influence biodiversity at the ecosystem level. However, microbial diversity within the fruiting zone (ON) is often reduced due to the strong dominance of the ring-forming fungus (Bonanomi et al., 2012; Li et al., 2022). The fungi within these rings actively participate in nutrient cycling through organic matter decomposition, promoting nutrient availability and improving soil fertility (Lindahl and Tunlid, 2015). They also assist plants in water and nutrient absorption, enhancing plant resilience under environmental stress (Bonfante and Genre, 2010). In degraded or disturbed ecosystems, the fungal mycelial network can help stabilize soil structure, reduce erosion, and facilitate vegetation recovery, thus contributing to ecological restoration (Cairney, 2005; Van Der Heijden et al., 1998). As a dominant saprotroph, *L. mongolica* plays a pivotal role in regulating matter and energy flow in grassland ecosystems, reflecting the intricate relationships between fungi and their environment (Bonanomi et al., 2012).

In recent years, research on *L. mongolica* has been primarily concentrated in the Inner Mongolia region, focusing on its population genetic structure, soil microbial community composition, and metabolic functions (Lu et al., 2018; Duan and Bau, 2021a; Wang et al., 2022; Duan et al., 2022a,b). In addition, extensive studies in alpine grassland ecosystems of the Tibetan Plateau have investigated fairy rings formed by related fungi such as *Floccularia luteovirens*, systematically revealing their genetic variation, metabolomic characteristics, and microbial community structures (Xing et al., 2017; Xing et al., 2018; Tang et al., 2024). However, studies on *L. mongolica* populations in Xinjiang remain very limited. As a marginal population located at the western edge of the species’ distribution, the Xinjiang population may possess unique ecological adaptations and microbe-fungus interaction patterns. Given the distinct habitat conditions in Xinjiang, including differences in climate, soil types, and vegetation composition compared to Inner Mongolia and the Tibetan Plateau, this population is likely to exhibit specific characteristics in fungal community structure, functional traits, and responses to environmental factors.

This study focuses on *L. mongolica* populations in the Bayanbulak grassland in Hejing County, Xinjiang, utilizing high-throughput sequencing technology in combination with traditional ecological survey methods. The research primarily addresses the following scientific questions: (1) What are the composition and structural characteristics of soil fungal communities within the fairy rings of the Bayanbulak grassland population? (2) What are the interaction patterns within the soil microbial communities of the fairy rings? and (3) How does *L. mongolica* influence key soil factors and the functional traits of fungal communities? This study aims to fill the knowledge gap regarding *L. mongolica* in this region and provide scientific basis for formulating regional conservation strategies.

## 2 Materials and methods

### 2.1 Study site

The study area is located in the Bayanbulak Grassland (Hejing County), a temperate steppe region renowned for its abundant fairy rings (Supplementary Table S1). The Bayanbulak Grassland is located between 42°10′N–43°30′N and 82°32′E–86°15′E, at the junction of the eastern, southern, and western Tianshan Mountains in the Xinjiang Uygur Autonomous Region, China. This region has a typical temperate continental arid climate, with a mean annual temperature of −4.8 °C and an average annual precipitation of approximately 276 mm (Lü et al., 2023). The study area is characterized primarily by typical alpine swamp meadows, with expansive alpine Stipa purpurea grasslands on mountain slopes and foothills. Moist valley bottoms support the development of swamp meadows and marsh vegetation dominated by *Carex*, *Kobresia*, and *Juncus* species (http://lcj.xinjiang.gov.cn, accessed March 15, 2025). During the fruiting period of fungi in August to September 2024, four *L. mongolica* fairy rings were collected (Supplementary Table S1).

### 2.2 Soil sampling and measurements

In August 2024, field sampling of *L. mongolica* and its associated fairy ring was conducted in the Bayanbulak grassland of Hejing County. During the sampling process, surface litter such as dead branches and leaves was carefully removed, and detailed records were made of each sampling site’s geographic coordinates, sampling time, and other relevant information. Based on the ecological characteristics of the fairy rings, each sampling site was divided into three representative zones (Supplementary Figure S1): the outside zone (OUT), located 5 meters away from the fruiting area and unaffected by fungal mycelia or associated vegetation; the fruiting zone (ON), corresponding to the area where fruiting bodies are present; and the inside zone (IN), situated at the geometric center of the ring structure.

A standardized sampling procedure was adopted in this study. For each of the four fairy rings, soil samples were collected from the OUT and ON zones using a five-point sampling method, while three parallel samples were collected around the central point of the IN zone. In total, 52 original soil samples were obtained (4 fairy rings × 13 sampling points). After sieving through a 2 mm mesh, samples from the same zone within each ring were thoroughly homogenized to form composite samples, resulting in 12 composite samples (4 fairy rings × 3 zones). To ensure analytical reliability, three biological replicates were established for each composite sample, yielding a total of 36 samples (12 composite samples × 3 replicates) for subsequent analyses. All samples were immediately flash-frozen in liquid nitrogen after collection and stored at −80 °C for subsequent physicochemical measurements and high-throughput sequencing.

### 2.3 Soil chemical and physical characterization

Soil pH was measured using a digital pH meter in a soil–water suspension at a ratio of 1:2.5 (w/v) (Guo et al., 2020). Soil moisture content was determined using the gravimetric (drying) method. Ammonium nitrogen (NH₄⁺-N) and nitrate nitrogen (NO₃^−^-N) were quantified using a fully automated Kjeldahl nitrogen analyzer. Total nitrogen (TN), total phosphorus (TP), and total potassium (TK) in the soil were determined following the method described by Gao et al. (2019). Soil organic carbon (SOC) and available potassium (AK) were measured using commercial assay kits according to the manufacturer’s instructions (Suzhou Keming Biotechnology Co., Ltd., Suzhou, China). Soil enzyme activities were measured using commercial assay kits provided by Grace Biotechnology (China). The specific enzymes included: soil sucrase activity (S-SC, Cat. No.: G0302W96), soil cellulase activity (S-CL, Cat. No.: G0308W), soil laccase activity (SL, Cat. No.: G0325W), soil urease activity (S-UE, Cat. No.: G0301W96), soil FDA hydrolase activity (FDA, Cat. No.: G0322W), and soil chitinase activity (SJ, Cat. No.: G0335W). All measurements were performed strictly according to the manufacturer’s instructions.

### 2.4 DNA extraction and PCR amplification

Genomic DNA was extracted from soil samples using the CTAB method (Miao et al., 2014). The purity and concentration of the extracted DNA were assessed by 1% agarose gel electrophoresis. Appropriate amounts of DNA were then transferred into sterile centrifuge tubes and diluted to a final concentration of 1 ng/μL using sterile water. PCR amplification of the ITS1 region was performed using primers ITS1-1F-F (5′-CTTGGTCATTTAGAGGAAGTAA-3′) and ITS1-1F-R (5′-GCTGCGTTCTTCATCGATGC-3′). All reactions were carried out in a 15 μL volume containing Phusion^®^ High-Fidelity PCR Master Mix with GC Buffer, 2 μM of each forward and reverse primer, and approximately 10 ng of template DNA. The PCR amplification was performed under the following conditions: initial denaturation at 98 °C for 1 min; 30 cycles of denaturation at 98 °C for 10 s, annealing at 50 °C for 30 s, and extension at 72 °C for 30 s; followed by a final extension at 72 °C for 5 min. The PCR products were mixed with an equal volume of 1X TAE buffer and electrophoresed on a 2% agarose gel. Target DNA bands were excised and purified using the Universal DNA Extraction Kit (TianGen, China) according to the manufacturer’s instructions.

### 2.5 Libraries generated and Illumina sequencing

Library preparation was performed using the NEB Next^®^ Ultra DNA Library Prep Kit. The constructed libraries were quality-checked with an Agilent 5,400 system and quantified by Q-PCR. Qualified libraries were sequenced on the Illumina platform. Raw sequences were processed through the DADA2 plugin in QIIME2 for quality filtered, denoised, merged, and non-chimeric to generate amplicon sequence variants (ASVs) (Callahan et al., 2016; Balvočiūtė and Huson, 2017; Caporaso et al., 2010). Representative ASV sequences were taxonomically annotated against the UNITE database version 8.2 (DeSantis et al., 2006).

### 2.6 Statistical analyses

All statistical analyses were performed using one-way ANOVA in IBM SPSS Statistics 27.0, with *p* < 0.05 considered statistically significant. Circos plots were generated using the R package “Circlize” to visualize the distribution of dominant fungal genera in the fairy rings of *L. mongolica* in Bayanbulak grassland. Potential microbial biomarkers were identified through linear discriminant analysis (LDA) effect size (LEfSe) method with an LDA score threshold >4.0, and the factorial Kruskal-Wallis test was set at *α* = 0.05 (Mandal et al., 2015; Segata et al., 2011). Alpha diversity metrics (Chao1, Shannon index, and Simpson’s reciprocal index) were calculated using the QIIME2 core-diversity plugin to assess within-sample diversity at the feature sequence level. Rarefaction curves of ASVs were generated in QIIME to evaluate sequencing depth adequacy. Inter-group comparisons of α-diversity indices were conducted using the Wilcoxon rank-sum test (IBM SPSS Statistics 27.0), with significance set at *p* < 0.05. Beta diversity was assessed using Bray-Curtis and weighted UniFrac distances to evaluate structural differences in microbial communities among samples, visualized through PCoA plots (Vázquez-Baeza et al., 2013). The statistical significance of beta diversity differences between groups was assessed using PERMANOVA (permutational multivariate analysis of variance). Co-occurrence networks were constructed and their topological properties analyzed using R packages “igraph,” “psych,” “WGCNA,” and “reshape2,” with network visualization performed in Gephi (Csardi, 2013; Revelle, 2023; Langfelder and Horvath, 2008; Wickham, 2020; Bastian et al., 2009). The Zi-Pi analysis of co-occurrence networks was conducted using R packages “ggplot2” and “igraph” and utilized Zi (range: 0–5) and Pi (range: 0–1) values to characterize node roles within the network (Han et al., 2022). Redundancy analysis (RDA) implemented in the “vegan” package was employed to explore potential relationships between microbial communities and environmental factors (Oksanen et al., 2013). PICRUSt2 and FUN-Guild were utilized to predict fungal community functions based on normalized ASV tables (Douglas et al., 2020; Nguyen et al., 2016). It should be noted that both methods rely on inference based on existing databases and algorithms, making them predictive analyses rather than experimentally verified results.

## 3 Result

### 3.1 Distinct diversity and assemblage structure of soil fungi across fairy ring zones of *Leucocalocybe mongolica*

**Figure 1:**
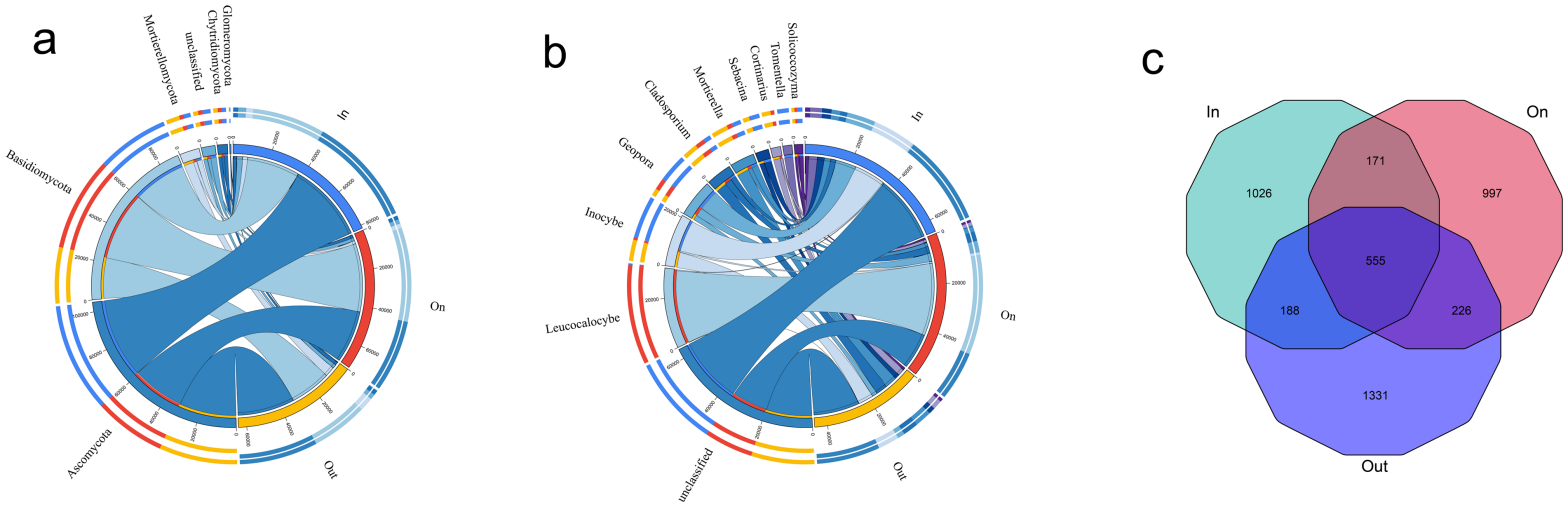
Fungal community composition analyses in different zones of *L. mongolica* fairy rings. **(a)** Phylum-level; **(b)** Genus-level; **(c)** Venn diagram of shared and unique ASVs across sample groups.

High-throughput sequencing of soils from three fairy ring zones 2,640,608 high-quality reads, corresponding to 12,371 ASVs. Taxonomic annotation based on the UNITE database revealed a high annotation rate at the genus level, and the rarefaction curve reached saturation, indicating sufficient sequencing depth (Supplementary Figures S2b,c). Community composition analysis showed that Ascomycota, Basidiomycota, and Mortierellomycota dominated across all zones (Figure 1a). Notably, the relative abundance of Basidiomycota in the ON zone reached 52.06%, higher than that in the IN (36.66%) and OUT zones (35.53%), representing a 1.4-fold increase compared to both peripheral zones (Supplementary Figure S3). At the genus level, *Leucocalocybe* was the most characteristic taxon of the fruiting zone, while *Inocybe*, *Geopora*, and *Cladosporium* were also important contributors (Figure 1b). The ON and OUT zones shared 226 ASVs, and the IN zone also shared a considerable number of ASVs with the ON (171) and OUT (188) zones. In addition, all three zones shared a substantial core microbiome comprising 555 ASVs. Notably, each zone contained a considerable number of unique ASVs (IN: 1026; ON: 997; OUT: 1331), reflecting distinct fungal community structures in different zones (Figure 1c). Despite a shared core microbiome of 555 ASVs, each zone harbored many unique taxa, indicating strong spatial differentiation of fungal communities shaped by *L. mongolica* activity.

**Figure 2:**
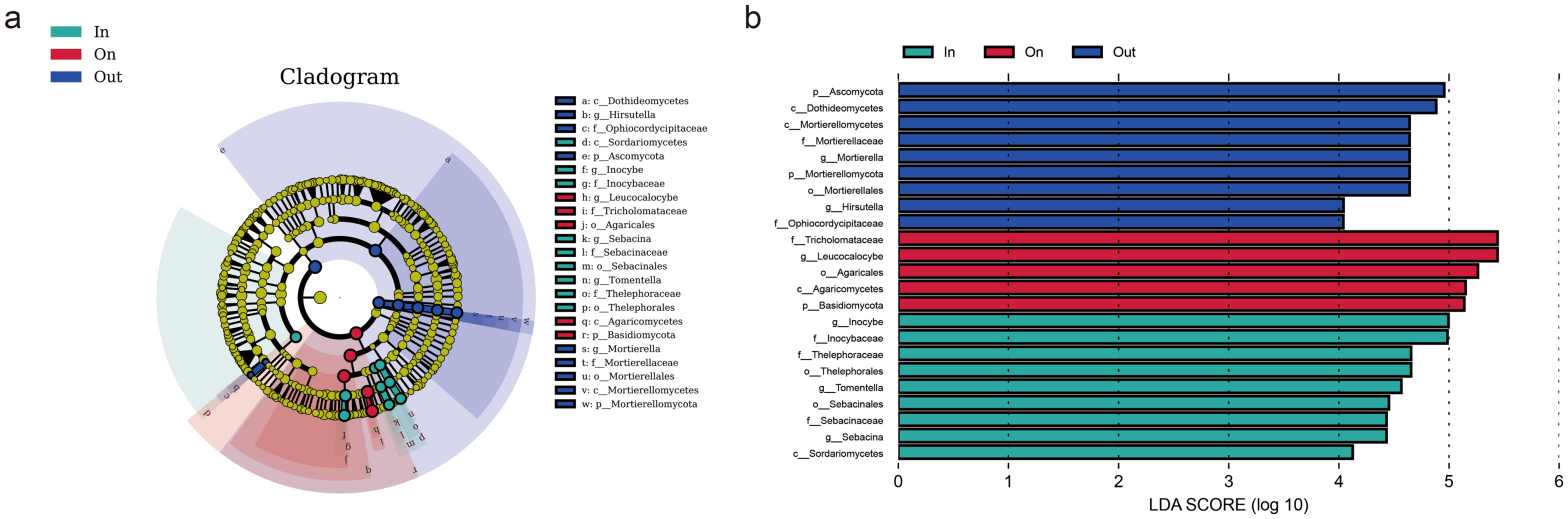
Comparison of differences in fairy ring soil fungi of *L. mongolica* sample plot (p: phylum; c: class; o: order; f: family; g: genus). **(a)** Cladogram is used to show taxonomic distribution of marker species in each group of samples; **(b)** Indicator microbial groups with LDA scores >4.0.

**Figure 3:**
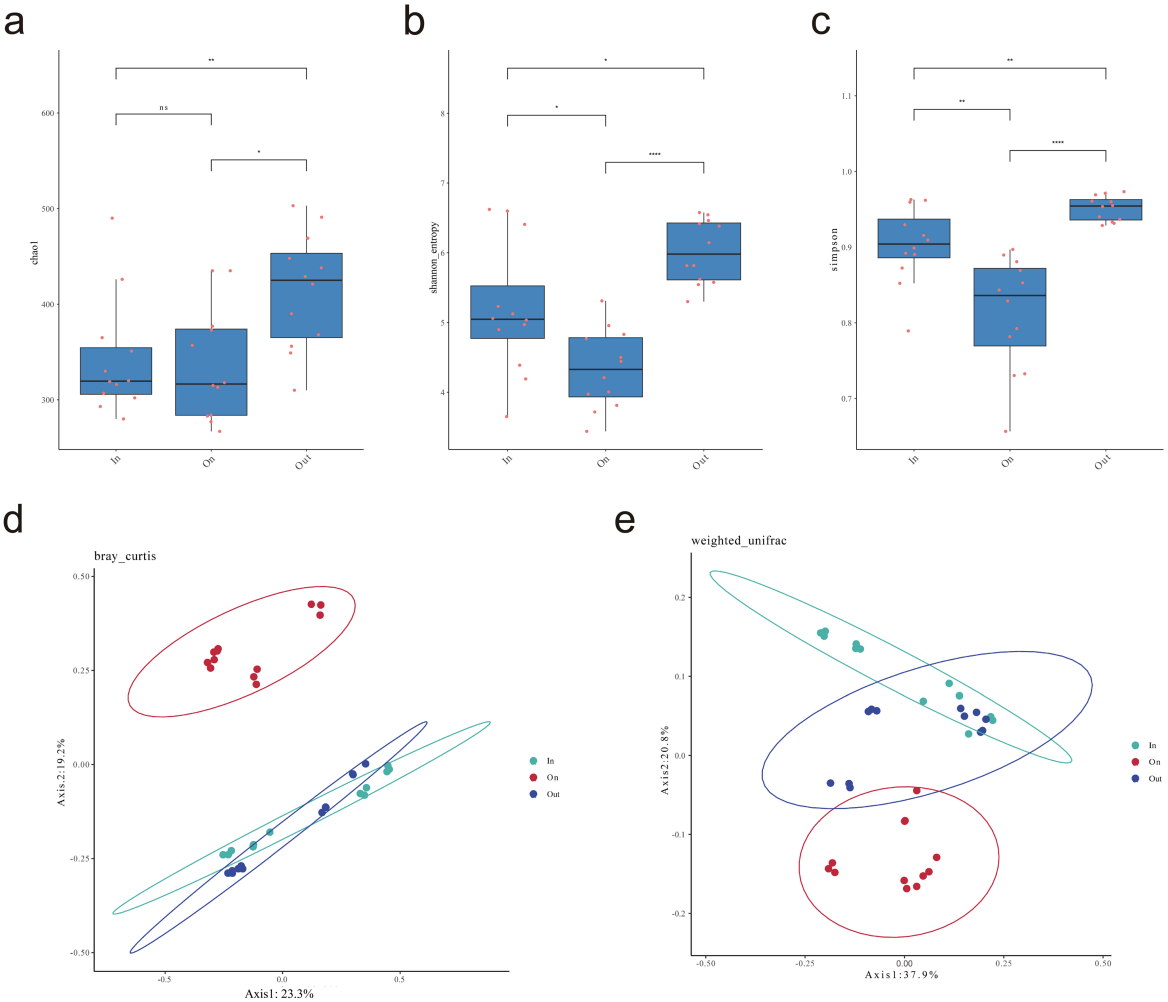
Analysis of soil fungal *α* and *β* diversity. **(a)** Chao1 index; **(b)** Shanon index; **(c)** Simpson index; **(d)** PCoA bray-curtis distance-based analysis **(e)** PCoA weighted UniFrac analysis. Using Wilcoxon rank sum test to analyze *p*-values. The “*” indicates a significant difference between the average values of two sets of samples, “*” represents *p* < 0.05, “**” *p* < 0.01, “***” *p* < 0.001. The statistical significance of beta diversity differences between groups was assessed using PERMANOVA.

LEfSe analysis identified distinct fungal biomarkers across zones, reflecting strong habitat filtering within fairy rings (Figure 2). The IN zone was enriched with Sordariomycetes and several saprotrophic taxa, suggesting enhanced decomposition potential. The ON zone was dominated by Agaricomycetes, particularly *Leucocalocybe*, highlighting its competitive advantage in the fruiting zone. In contrast, the OUT zone supported diverse Ascomycota and Mortierellomycota lineages, including *Mortierella* and *Hirsutella*, indicating broader ecological strategies. Overall, these patterns demonstrate that *L. mongolica* activity drives sharp community differentiation through selective pressures in different microenvironments. Diversity analyses revealed clear spatial patterns: fungal richness and evenness were highest in the OUT zone, intermediate in the IN zone, and lowest in the ON zone (*p* < 0.05), indicating that *L. mongolica* activity suppresses community diversity within the fruiting zone (Figures 3a–c). Beta-diversity analysis further showed strong separation among zones (*p* < 0.01), with ON samples forming distinct clusters (Figures 3d,e). These patterns suggest that *L. mongolica* shapes fungal assemblages through microenvironmental modification and nutrient competition, reinforcing niche differentiation within fairy rings (Figure 3).

### 3.2 Co-occurrence network of soil fungi

**Figure 4:**
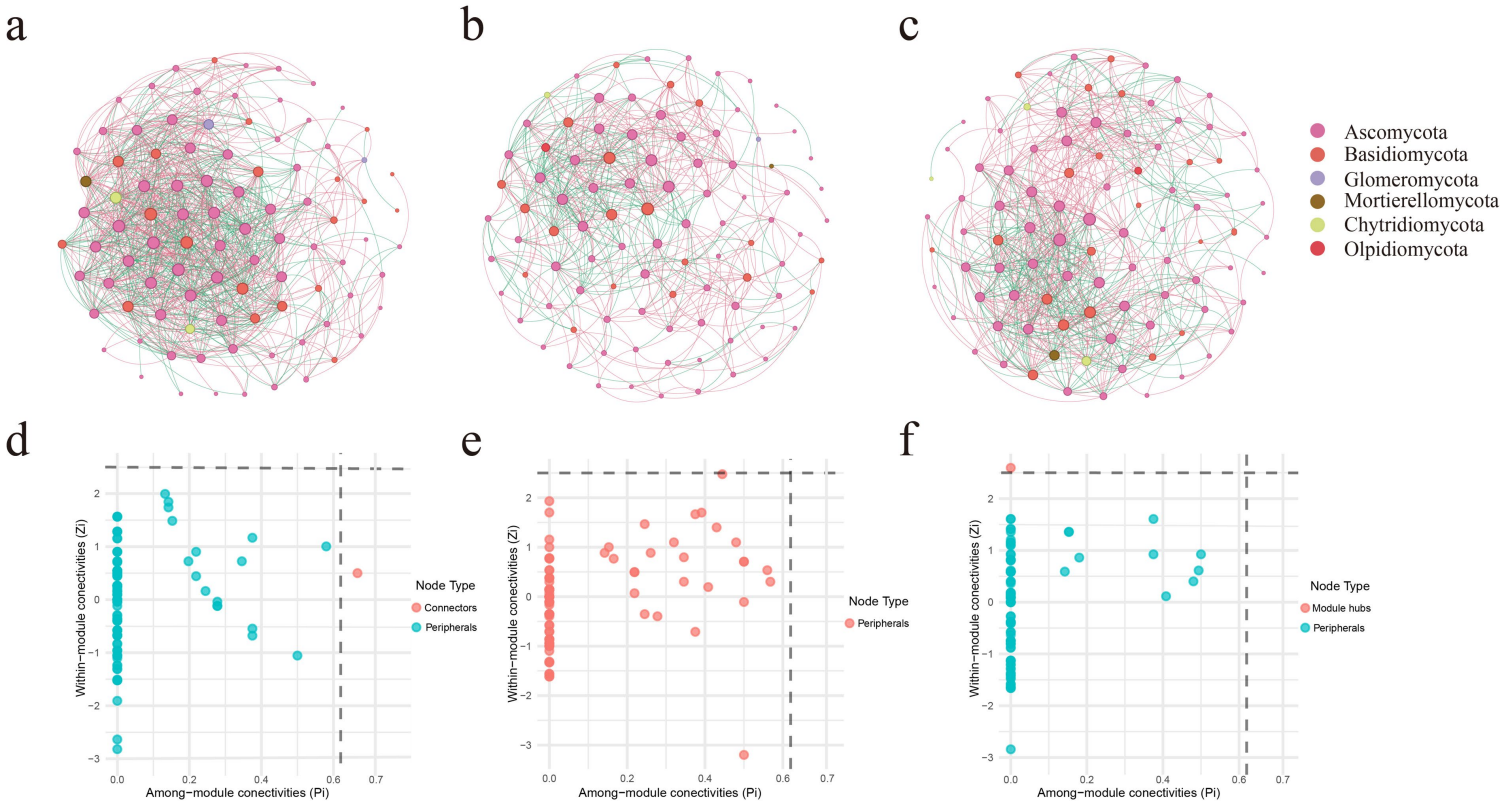
Co-occurrence network pattern of three different zones of *L. mongolica* fairy rings. **(a–c)** Represent the inside zone (IN), the fruiting zone (ON), and the outside zone (OUT), respectively. **(d–f)** Represent Zi-Pi graphs based on topological roles in networks of IN, ON, and OUT, respectively.

Based on co-occurrence network analysis, this study revealed distinct interaction patterns of soil fungal communities across different zones of *L. mongolica* fairy rings (Supplementary Table S2; Figure 4). The network in the IN zone comprised 196 nodes and 1,241 edges, exhibiting the highest connectivity density (average degree = 12.663) and clustering coefficient (0.533). This indicates a highly interconnected fungal community with relatively low modularity (0.268), suggesting a more complex network structure and a higher level of symbiotic relationships in this zone. In contrast, the ON zone network (201 nodes, 770 edges) exhibited distinct topological characteristics, with the lowest average degree (7.662) and clustering coefficient (0.433), but the highest modularity (0.416), indicating a more specialized and clearly partitioned interaction pattern dominated by *L. mongolica*. The OUT zone network (198 nodes, 945 edges) showed intermediate features, with an average degree (9.545) and modularity (0.378) falling between those of the IN and ON zones. In addition, the ASVs directly associated with *L. mongolica* included *Thelebolus*, *Coprinellus*, *Penicillium*, *Claroideoglomus*, and *Chalara* (|r| > 0.7, *p* < 0.05). Among these, only *Thelebolus* exhibited a positive interaction with *L. mongolica*, while the other four genera showed negative interactions. Notably, *Coprinellus* displayed strong positive associations with *Penicillium*, *Claroideoglomus*, and *Chalara*.

By calculating within-module connectivity (Zi) and among-module connectivity (Pi), we identified potential keystone species (Figures 4d–f). Ascomycota and Basidiomy-cota were the key fungal phyla across the three zones of the fairy ring. The ON zone exhibited a homogenized network structure, characterized by all ASVs being classified as peripheral nodes. In the IN zone, one ASV was identified as a connector, affiliated with the phylum Ascomycota and the genus *Elasticomyces*. In the OUT zone, one ASV was identified as a module hub, affiliated with the phylum Basidiomycota and the genus *Hyphodontia*. These keystone nodes in the IN and OUT zones may contribute to a higher level of ecological functional coordination.

### 3.3 Relationship between soil factors and fungal communities

The soil properties in different zones of *L. mongolica* fairy rings exhibit significant spatial heterogeneity (Supplementary Table S3). Notably, the ON zone displays unique physicochemical characteristics, with significantly lower soil pH compared to the In and OUT zones (*p* < 0.05), as well as elevated levels of ammonium and nitrate nitrogen. The OUT zone is characterized by optimal total nutrient levels, particularly with significantly higher total potassium and available potassium contents than the other zones (*p* < 0.05), while it has the lowest organic carbon content. In contrast, the IN zone exhibits the highest organic carbon content but relatively lower potassium levels. This heterogeneous nutrient distribution reflects the ecological functional differentiation among various zones of the fairy rings. Enzyme activity analysis revealed that the OUT zone exhibited significantly higher activities of soil cellulase, urease, FDA hydrolase, and chitinase compared to the ON zone (*p* < 0.05), where chitinase activity was the lowest among the three zones. These differences suggest that *L. mongolica* may selectively modulate soil enzyme activities by suppressing certain hydrolases while promoting cellulose degradation processes, thereby reshaping the soil functional profile within the ON zone. This finding provides important insight into the underlying mechanisms of fairy ring formation.

**Figure 5:**
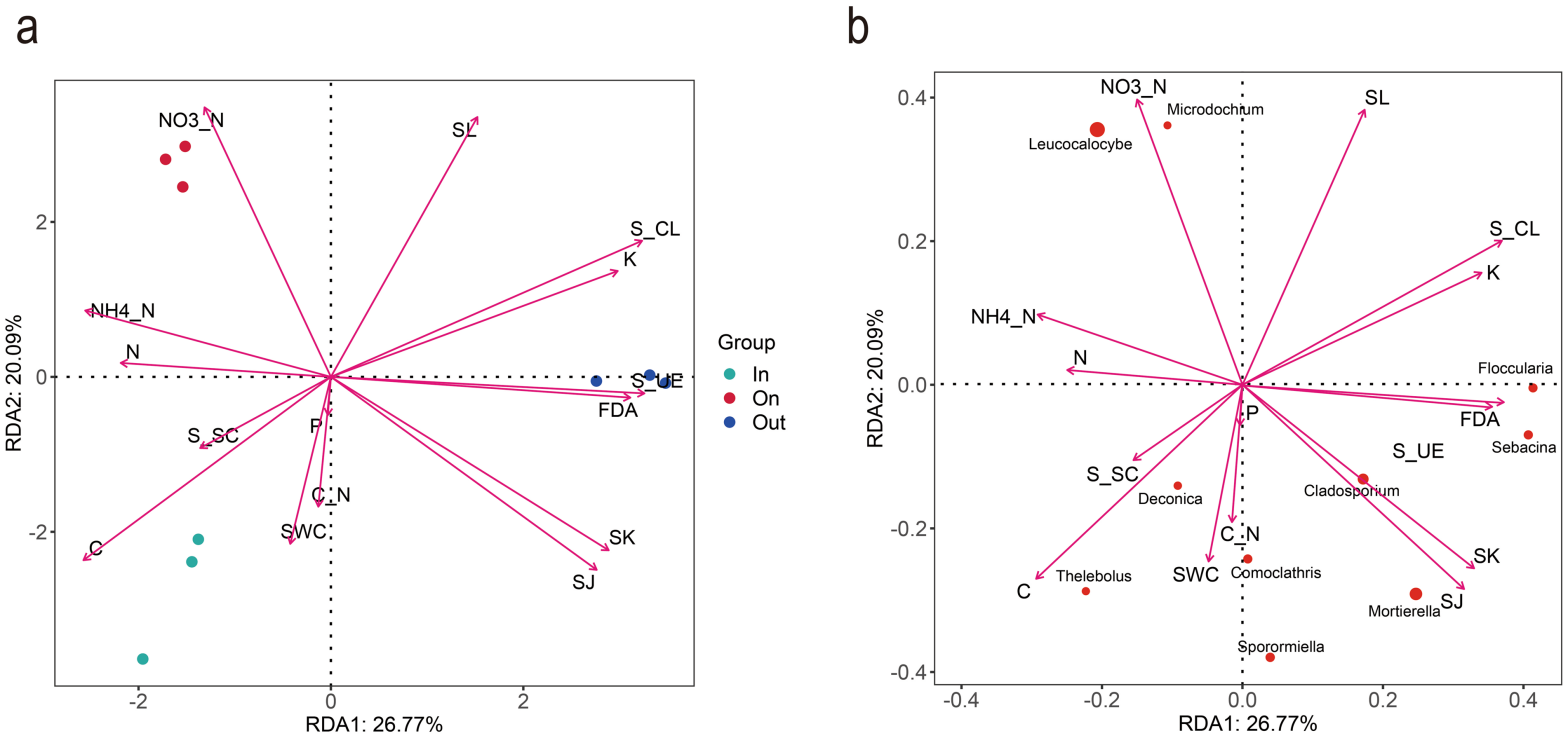
RDA ordination of fungal community-environment interactions. **(a)** Fungal genera distribution across zones (IN/ON/OUT) in relation to soil factors (RDA1: 26.77%; RDA2: 20.09%). **(b)** Top 10 genera in the ON zone and their correlations with soil properties.

RDA further revealed functional differentiation among different zones of *L. mongolica* fairy rings in the Bayanbulak grassland. The first and second RDA axes explained 26.77 and 20.09% of the total variation, respectively (Figure 5), indicating that the model effectively captured the main environmental driving factors. The distinct separation of sample groups in the RDA ordination plot suggests significant differences in fungal community structure across the different zones of the *L. mongolica* fairy rings (permutation test, *p* < 0.01). Notably, the fungal community abundance in the fruiting zone was significantly positively correlated with ammonium nitrogen, nitrate nitrogen, and soil laccase activity, while showing a significant negative correlation with available potassium. This suggests potential nutrient competition between the fungal community and plants in nitrogen transformation and lignin degradation processes within this zone. In contrast, the fungal community in the out zone exhibited distinct enzymatic activity patterns, being positively correlated with urease and cellulase activities but negatively correlated with soil nitrogen content. This may reflect functional differentiation of microbial carbon and nitrogen metabolism in this zone. RDA of the top 10 dominant fungal genera further clarified that *L. mongolica* and *Microdochium* were strongly associated with soil ammonium nitrogen, nitrate nitrogen, and laccase activity, further highlighting the relationship between *L. mongolica* and ammonium-nitrate nitrogen and laccase activity.

### 3.4 Functional prediction of soil fungi

**Figure 6:**
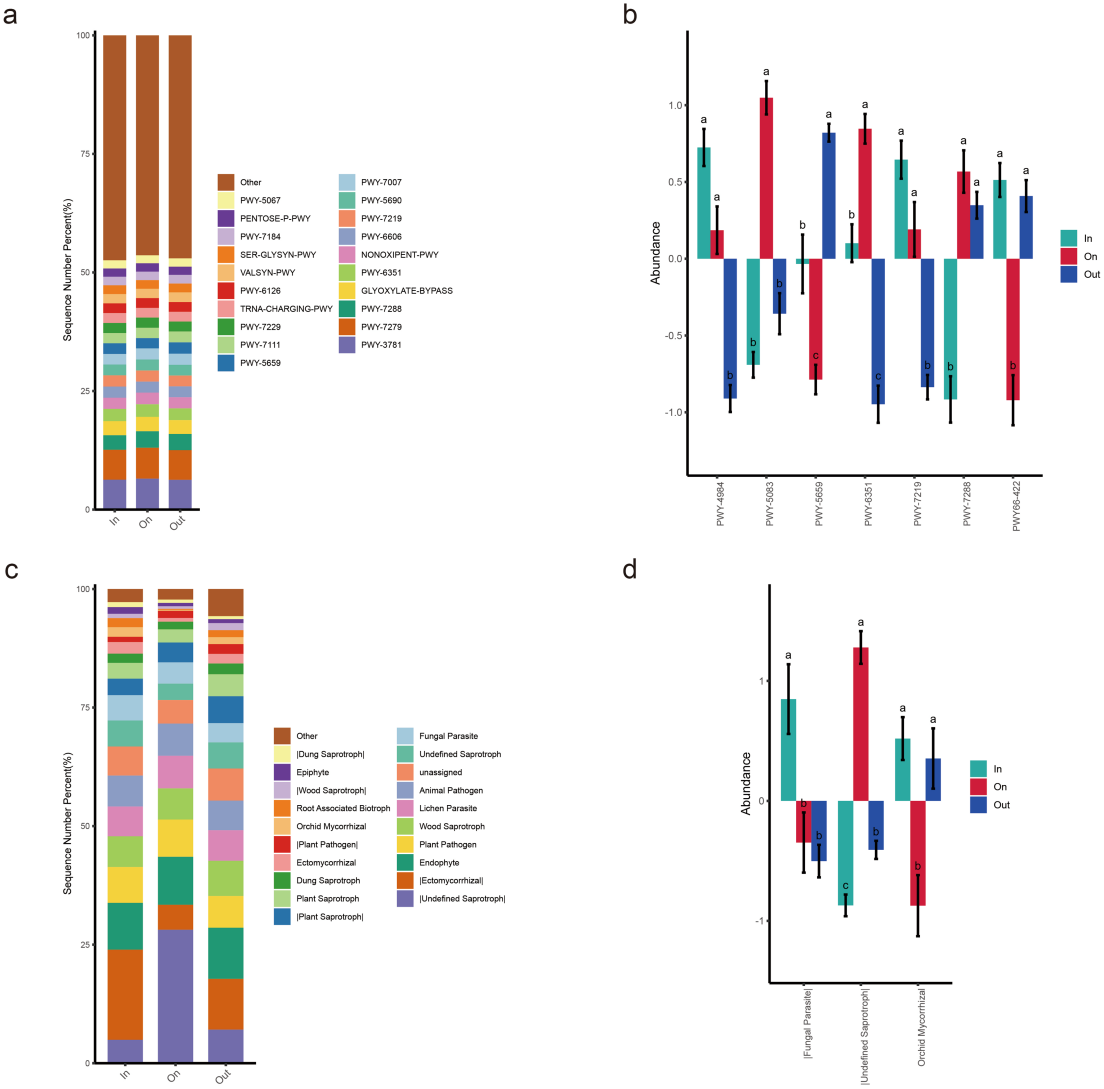
Functional prediction analysis of soil fungi based on PICRUSt2 **(a,b)** and FUNGuild **(c,d)**. **(a)** Relative abundance distribution of fungal metabolic pathways predicted by PICRUSt2. **(b)** All significantly different MetaCyc pathways (*p* < 0.05) identified by ANOVA and Duncan’s test. **(c)** Relative abundance distribution of fungal metabolic pathways predicted by FUNGuild. **(d)** All significantly different functional guilds (*p* < 0.05) identified by ANOVA and Duncan’s test.

In this study, PICRUSt2 was employed to predict the functional profiles of MetaCyc metabolic pathways in soil fungal communities across three zones (Figures 6a,b). The results revealed that PWY-3781 (aromatic compound degradation pathway) and PWY-7279 (branched-chain amino acid biosynthesis pathway) were the common dominant metabolic pathways shared among the three zones, with their relative abundances consistently around 6% (Figure 6a). This indicates the conserved nature of these fundamental metabolic functions within soil fungal communities across different geographic locations. Multiple comparison analysis of the bar chart revealed that the ON zone exhibited significant site-specificity in certain metabolic pathways. Specifically, the abundances of PWY-5083 (polycyclic aromatic hydrocarbon degradation pathway) and PWY-6351 (chitin degradation pathway) were significantly higher than those in the other two zones (*p* < 0.05) (Figure 6b). In contrast, the abundances of PWY-5659 (carotenoid biosynthesis pathway) and PWY-66-422 (siderophore biosynthesis pathway) were significantly lower in the ON zone compared to other zones. These results suggest that fungi in the ON zone may employ distinct metabolic strategies to cope with oxidative stress and iron limitation.

Functional prediction analysis based on FUNGuild (Figures 6c,d) revealed significant functional differentiation of soil fungal communities across different zones of the fairy ring. The ON zone exhibited distinct functional guild characteristics, with the relative abundance of Undefined Saprotrophs reaching 28.13%, which was significantly higher than those in the IN and OUT zones (*p* < 0.05) (Figure 6c). In contrast, the IN and OUT zones were dominated by the Ectomycorrhizal guild, with mean relative abundances of 18.9 and 10.7%, respectively. Notably, the abundances of four nutritional functional groups (Plant Pathogen, Wood Saprotroph, Lichen Parasite, and Animal Pathogen) remained consistently high across all zones. Specifically, the abundance of Orchid Mycorrhizal fungi in the ON zone was significantly lower than that in the IN and OUT zones (*p* < 0.05) (Figure 6d).

## 4 Discussion

### 4.1 Fungal community structure and ecological strategies in *Leucocalocybe mongolica* fairy rings

Distinct fungal community structures were observed among different zones of the fairy ring (Figure 1). The relative abundance of Basidiomycota in the ON zone reached 52.06%, which was significantly higher than that in the IN zone (36.66%) and the OUT zone (35.53%) (Supplementary Figure S3). This dominance was primarily driven by *Leucocalocybe* (family Tricholomataceae), whose LDA score exceeded 4.0, indicating that this species serves as a key biomarker for the ON zone. In addition, this study identified *Cladosporium* as another dominant fungal genus in the soil of *L. mongolica* fairy rings (Supplementary Figure S3b). Previous research has shown that secondary metabolites secreted by *Cladosporium*, including gibberellins, can promote plant growth (Răut et al., 2021). Therefore, we can speculate that *Cladosporium* plays a positive role in the formation of fairy rings and plant growth. The metabolites of *Cladosporium* interact beneficially with plants, while exhibiting antagonistic effects by inhibiting the germination and growth of several other fungal species (Moricca et al., 2005; Moricca et al., 2001).

*F. luteovirens* populations exhibit notable genetic variation and phylogenetic differentiation across altitudinal gradients (Xing et al., 2017). Moreover, microbial investigations revealed that its fairy rings harbor unique microbial communities and potential mycorrhiza-helper bacteria that facilitate plant growth (Xing et al., 2018). Recent metabolomic profiling further indicated geographical variation in secondary metabolites, which may contribute to the antioxidative capacity and ecological adaptation of *F. luteovirens* (Tang et al., 2024). In contrast, our study showed that the fruiting zone of *L. mongolica* fairy rings in the Bayanbulak grassland was characterized by sharply reduced fungal diversity, simplified microbial networks, and elevated laccase activity, highlighting a predominantly saprotrophic regulation strategy. This contrasts with the ectomycorrhizal symbiosis-based mechanisms reported for *F. luteovirens*. Such differences suggest that variation in ecological strategies between saprotrophic (*L. mongolica*) and ectomycorrhizal (*F. luteovirens*) fungi drives divergent pathways of nutrient competition, niche modification, and microbial interactions.

The Venn diagram analysis highlighted that although the three zones shared a substantial core microbiome (555 ASVs), each zone also harbored a large number of unique ASVs. This indicates that the fungal communities are strongly structured by spatial heterogeneity within the fairy ring ecosystem. The coexistence of a shared core microbiome and many zone-specific taxa suggests that while some fundamental ecological functions are maintained across zones (Duan and Bau, 2021a), distinct environmental conditions and fungal–plant interactions drive the differentiation of community assemblages in each zone. The high abundance of *Leucocalocybe* indicates its important role in maintaining the fundamental ecological functions of the fairy rings. Notably, the greater number of shared ASVs between the ON and OUT zones align with their spatial proximity observed in the PCoA analysis (Figures 2d,e), suggesting a certain degree of connection between the fungal communities in these two areas. Alpha diversity revealed the trend OUT > IN > ON (Figures 2a–c), consistent with previous reports (Wang et al., 2022; Duan et al., 2022a; Duan and Bau, 2021b; Kim et al., 2013; Ito et al., 2017), while *β*-diversity confirmed clear spatial separation among zones (Figures 2d,e), highlighting the strong influence of *L. mongolica*. Its vigorous mycelial growth preferentially exploits soil carbon and nitrogen sources, particularly by occupying the rhizosphere niche, effectively limiting the access of other fungi to nutrients and thereby suppressing their growth, resulting in a competitive exclusion effect (van Der Heijden et al., 2015; Liu et al., 2023; Liu et al., 2024). Furthermore, the mechanisms underlying these ecological dynamics primarily involve the secretion of inhibitory secondary metabolites (Keller, 2019; Bennett et al., 2017), and physical alteration of soil microhabitats (van Der Heijden et al., 2015). Together, these processes promote competitive exclusion and shape the unique fungal assemblage in the ON zone.

### 4.2 Topological features of microbial networks reveal the community restructuring mechanism of *Leucocalocybe mongolica*

The ON zone network showed higher modularity (0.416) but lower connectivity density (average degree 7.662) compared to the other zones, indicating reduced efficiency despite a compact core structure. These topological traits align with the low Shannon diversity (Figures 2a–c), *β*-diversity clustering (Figures 2d–f), and saprotroph dominance (Figure 6), suggesting that *L. mongolica* reshapes soil microecology by restructuring the fungal interaction network. In the co-occurrence network analysis, we further examined the ASVs directly associated with *Leucocalocybe mongolica* and their interaction patterns with other ASVs in the network. The results showed that *Thelebolus* exhibited a positive interaction with *L. mongolica*. Many species of *Thelebolus* are psychrophilic and display strong adaptability to cold and other extreme environments (Wicklow and Malloch, 1971; Mi et al., 2024). This characteristic suggests that *Thelebolus* may form a mutually beneficial relationship with *L. mongolica* within the fungal community, thereby enhancing their adaptive capacity and niche expansion potential in the soil environment. On the other hand, *Coprinellus* exhibited a negative interaction with *L. mongolica*, which may be attributed to the fact that both belong to Basidiomycota and are saprotrophic fungi (Hussain et al., 2018), thereby competing for similar soil nutrient resources. *Claroideoglomus*, as an arbuscular mycorrhizal fungus (Wu et al., 2024), may also compete with *L. mongolica* for root colonization sites or host-derived nutrients. Notably, *Coprinellus* showed strong positive associations with *Penicillium*, *Claroideoglomus*, and *Chalara*. This phenomenon may suggest that these taxa form functional cooperation or niche complementarity to cope with the environmental pressures imposed by *L. mongolica*. Such findings provide new insights into the assembly mechanisms of fungal communities under the influence of *L. mongolica*.

Combined with the network topology characteristics, it can be further inferred that *L. mongolica* may reshape the local microbial interaction network through three pathways. First, its intense resource competition (Morriën et al., 2017) likely eliminates numerous sensitive species, resulting in a reduction in network connectivity. Secondly, secondary metabolites secreted by fungi in the fairy ring may selectively promote the growth of specific tolerant species, forming more specialized interaction modules (Bennett et al., 2017). Third, *L. mongolica* plays a central role in fungal community assembly by establishing synergistic relationships with tolerant taxa (such as *Thelebolus*) to maintain community stability, while simultaneously leveraging competitive exclusion mechanisms to suppress the expansion of competing groups. Notably, no keystone taxa were detected in the ON-zone network, with all ASVs functioning as peripheral nodes. This phenomenon supports the “top-down” control hypothesis of *L. mongolica* as an absolute dominant species on community structure (Morriën et al., 2017), whereby it simplifies the network by suppressing interactions among other species and forming a self-centered network.

### 4.3 Nitrogen enrichment and laccase activity synergy in *Leucocalocybe mongolica* fairy rings

The fairy ring soil of *L. mongolica* exhibited elevated levels of ammonium and nitrate, consistent with previous studies (Duan et al., 2022a; Liu et al., 2024). Nitrogen is an essential nutrient for large fungi during mycelial growth and fruiting body development, as it is required for the biosynthesis of key cellular components such as proteins and nucleic acids (Kuypers et al., 2018; Carrasco and Preston, 2020). The lower pH observed in the fairy ring soil promotes the mineralization of organic nitrogen into NH₄⁺, while the increased NH₄⁺ concentration stimulates the proliferation of nitrifying microorganisms, thereby enhancing nitrogen utilization (Liu et al., 2024). In addition, unlike previous studies (Duan et al., 2022a), the cellulase activity in the soil of the fairy ring was significantly lower than that in the outer area, whereas laccase activity was significantly higher than in the other two areas. Laccase can catalyze the biodegradation of lignin and humic substances, thereby promoting the cycling of soil organic matter (Schuetz et al., 2014; Zavarzina et al., 2018). Aromatic compounds produced from lignin degradation may be utilized by certain nitrifying bacteria, thereby enhancing the nitrification process (Zheng et al., 2024). This could partly explain the higher nitrate nitrogen levels observed in the fairy ring zone. RDA indicated that the fungal community in the ring zone was significantly positively correlated with ammonium nitrogen, nitrate nitrogen, and laccase activity (Figure 5), further clarifying the relationship between environmental factors and fungal community composition. It should be noted that the physicochemical properties and enzyme activities in this study were measured in one representative fairy ring, whereas the fungal community sequencing was conducted across four independent rings. This design was adopted primarily due to resource constraints. Nevertheless, the observed patterns of nutrient availability and enzyme activities are consistent with previous reports on *L. mongolica* and other fairy ring fungi, and the inclusion of three biological replicates per zone ensures statistical reliability. We acknowledge that extending physicochemical and enzymatic analyses to multiple fairy rings would further enhance the generality of the results, and this represents an important direction for future research.

These fungus-driven nutrient changes have important ecological implications for grassland management and restoration. *L. mongolica* can enhance soil available nitrogen and serve as a natural biofertilizer for degraded grasslands; inoculation with such functional fungi can accelerate nutrient cycling, improve soil fertility, and promote vegetation recovery. Its elevated laccase activity enhances the decomposition of recalcitrant organic matter, facilitating organic matter turnover and nutrient release. This suggests that cultivating fungal communities with high lignin-degrading capacity could be an effective strategy to accelerate ecosystem restoration. Therefore, fairy ring–forming fungi not only provide ecological functions but also represent valuable biotic agents for sustainable grassland management, with microbial functions being key to restoring ecosystem productivity and stability.

### 4.4 Functional divergence and ecological adaptation of soil fungal communities in *Leucocalocybe mongolica* fairy rings

This study systematically elucidates the functional differentiation characteristics and ecological adaptation strategies of soil fungal communities in different zones of the *L. mongolica* fairy rings through MetaCyc metabolic pathway analysis and FUN-Guild functional prediction. The metabolic pathway analysis revealed that fundamental metabolic functions, such as PWY-3781 (aromatic compound degradation) and PWY-7279 (branched-chain amino acid biosynthesis), remained relatively stable across the three zones (6%), reflecting the conservatism of core metabolic functions in soil fungi. Notably, the enrichment of polycyclic aromatic hydrocarbon degradation (PWY-5083) and chitin degradation (PWY-6351) pathways observed in the ON zone (*p* < 0.05) corresponds with the high soil laccase activity detected in this area. Furthermore, a decrease in the siderophore biosynthesis pathway (PWY66-422) was found in the ON zone. Siderophores, produced by most fungal species, are iron-chelating agents involved in high-affinity iron uptake and intracellular processing (Happacher et al., 2023). The reduced siderophore biosynthesis pathway in this zone corroborates its lowest fungal diversity. Fungi in the ON zone may cope with oxidative stress and iron limitation through alter-native mechanisms, such as hyphal network transport, reflecting adaptive evolution to this unique microenvironment.

FUNGuild functional prediction further confirmed this functional differentiation. The high abundance of undefined saprotrophs (Figure 6c) in the ON zone indicates their significant role in organic matter decomposition and nutrient cycling (Liers et al., 2010; Allmér et al., 2009). This result is consistent with the enhanced organic matter degradation metabolic traits observed in the fairy ring zone, suggesting that *L. mongolica* may optimize carbon source utilization efficiency by reshaping saprotrophic functional groups. In contrast, the predominance of ectomycorrhizal fungi in the In and Out zones (Figure 6c) reflect a plant-fungal interaction network closer to the natural state in these areas (Tedersoo et al., 2020). Notably, the significantly reduced abundance of orchid mycorrhizal fungi in the ON zone (Figure 6d) may originate from allelopathic inhibition by *L. mongolica*, a phenomenon consistent with the allelopathic niche competition mechanism among fungi (Bennett et al., 2017). Such a synergistic “enzymatic degradation–allelopathy” interaction offers novel insights into the ecological competitive advantages of large fungi. It should be noted that functional annotations obtained from these two prediction approaches may involve certain biases, particularly given the incomplete coverage of fungal functional databases. Future studies should integrate metagenomics, transcriptomics, or enzymatic activity assays to more accurately elucidate the actual functions of fungal communities.

## 5 Conclusion

This study systematically elucidated the structure, diversity, and ecological functions of soil fungal communities across different zones of *L. mongolica* fairy rings in the Bayanbulak Grassland, Xinjiang. Our findings suggest that *L. mongolica* may establish a metabolic advantage in the fruiting zone by enhancing laccase activity and regulating nitrogen availability, which in turn could reshape microbial networks and suppress competing functional groups. Notably, the observed association between elevated laccase activity and nitrogen cycling in fairy ring soils provides preliminary evidence of a potential mechanism through which macro fungi influence belowground functional zonation.

These findings not only deepen ecological understanding but also hold direct practical significance for management. Protecting fungal diversity and the microenvironments of fairy rings—through measures such as limiting overgrazing and minimizing human disturbance—may help sustain ecosystem functions. Moreover, the functional roles of *L. mongolica* and its symbiotic fungi in nutrient cycling and plant stress tolerance suggest strong potential for ecological restoration of degraded grasslands, for instance by promoting natural expansion or assisted establishment of fairy rings. Considering the unique climatic and edaphic conditions of Xinjiang, region-specific monitoring and adaptive management will be essential to ensure effective conservation outcomes. Future work integrating metagenomics and metabolomics will be essential to unravel the molecular basis of these interactions and to translate them into practical restoration measures. Such efforts should also consider the ecological balance between *L. mongolica* and the associated microbial communities, to achieve synergistic protection of symbiotic microbes and plant functional groups (Nilsson et al., 2019).

## Data Availability

The datasets presented in this study are publicly available. This data can be found here: https://www.ncbi.nlm.nih.gov/, BioProject accession number PRJNA1335383, and SRA accession range SRR35646027 – SRR35646062.
