# Supplementary material for: Soil fungal community structure and ecological functions in fairy rings of Leucocalocybe mongolica in Bayanbulak grassland
Source: Front Microbiol. 2025 Oct 14;16:1667514. doi: 10.3389/fmicb.2025.1667514 (PMC12558830; doi:10.3389/fmicb.2025.1667514)
Supplement: Supplementary file 1 [file Supplementary_file_1.docx]

Supplementary Material

# Supplementary Figures and Tables

## Supplementary Figures


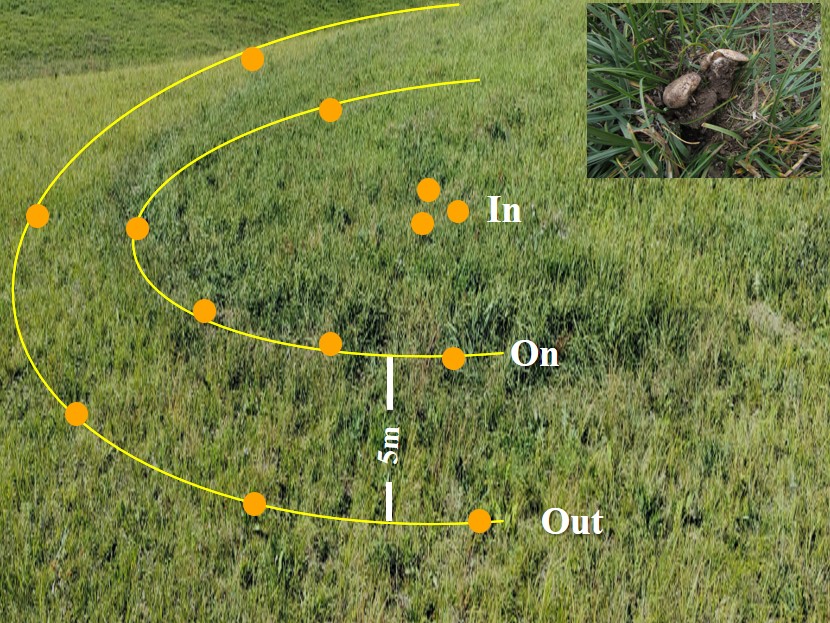


**Supplementary Figure 1.** Sampling schematic of *L. mongolica* fairy rings and morphological characteristics of basidiocarps.


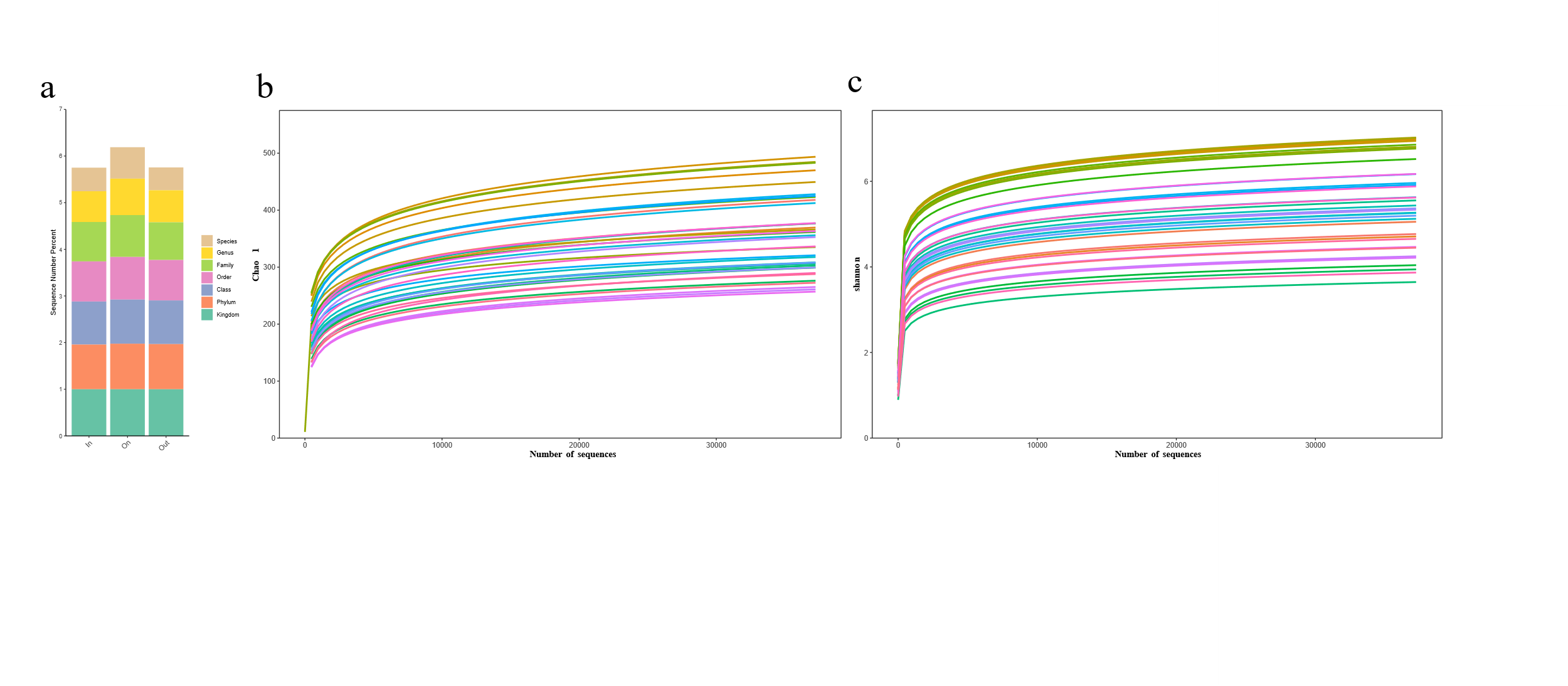
**Supplementary Figure 2.** ASV annotation at different taxonomic levels and rarefaction curves of various diversity indices. (**a**) Bar plot showing sequence annotation rates at different taxonomic levels across samples. The rarefaction curves of (**b**) Chao1 index and (**c**) Shannon index.


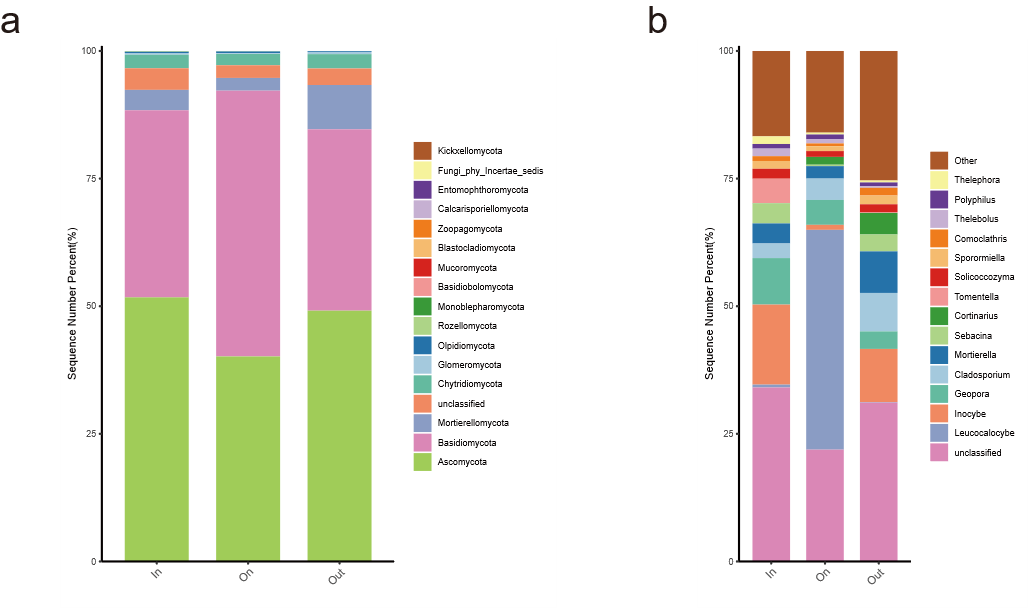


**Supplementary Figure 3.** Bar plots showing relative taxonomic distributions at phylum and genus levels across sample groups (a) Phylum; (b) Genus.

## Supplementary Tables

**Supplementary Table 1.** Geographic coordinates of fairy rings formed by *L mongolica*.

| **Sites** | **Site code** | **Longitude** | **Latitude** | | **Altitude (m)** |  |
| --- | --- | --- | --- | --- | --- | --- |
|  |  |  |  |  |  |  |
| Hejing County | FR1 | 86°2′10.3834″ | 43°7′49.5012″ | 3213.65 | |  |
| Hejing County | FR2 | 86°1′18.9192″ | 43°9′47.3311″ | 3031.31 | |  |
| Hejing County | FR3 | 86°3′39.3909″ | 43°4′40.4332″ | 3310.13 | |  |
| Hejing County | FR4 | 86°4′20.5641″ | 43°5′42.5432″ | 3125.15 | |  |

**Supplementary Table 2.** Network topological parameters of soil fungal communities across different fairy ring zones.

|  | **Nodes** | **Edges** | **Network diameter** | **Average degree** | **Average Path Length** | **clustering coefficient** | **Modularity** |
| --- | --- | --- | --- | --- | --- | --- | --- |
| IN | 196 | 1241 | 6 | 12.663 | 2.896 | 0.533 | 0.268 |
| ON | 201 | 770 | 5 | 7.662 | 3.103 | 0.433 | 0.416 |
| OUT | 198 | 945 | 6 | 9.545 | 3.016 | 0.477 | 0.378 |

**Supplementary Table 3.** Soil physical and chemical properties of soil samples from the fairy ring of *L mongolica*. One-way ANOVA was conducted, followed by multiple comparisons using Tukey’s HSD method. Statistically significant difference was indicated by different letters when *p*< 0.05.

|  | **IN** | **ON** | **OUT** |
| --- | --- | --- | --- |
| pH | 7.82±0.04a | 7.42±0.03c | 7.64±0.03b |
| SWC (%) | 13.39±0.01a | 12.49±0.19b | 12.75±0.33ab |
| NH_4_^+^-N (mg kg-1) | 2.38±0.07c | 2.53±0.12a | 2.01±0.13b |
| NO_3_^-^-N (mg kg-1) | 10.90±0.19c | 14.99±0.11a | 11.60±0.04b |
| TN (%) | 0.59±0.02ab | 0.60±0.01a | 0.56±0.00b |
| TP (g kg-1) | 1.02±0.03b | 0.99±0.001 | 1.00±0.01a |
| TK (g kg-1) | 2.88±0.04b | 2.96±0.003b | 3.07±0.015a |
| SOC (g kg-1) | 5.89±0.04a | 5.5±0.04b | 5.3±0.01c |
| AK (g kg-1) | 0.37±0.002b | 0.35±0.003c | 0.38±0.0005a |
| S-SC (μg d-1 g-1) | 128.91±1.58 | 125.07±2.56 | 122.63±1.07 |
| S-CL (μg d-1 g-1) | 1792.54±51.60c | 2811.87±49.61b | 3892.94±65.25a |
| SL (μg d-1 g-1) | 13.40±0.49c | 70.56±0.71a | 64.14±0.66b |
| S-UE (μg d-1 g-1) | 2086.03±24.29b | 2079.87±22.88b | 2310.87±28.45a |
| FDA (μg d-1 g-1) | 445.98±16.25b | 443.83±16.94b | 539.80±2.83a |
| SJ (μg d-1 g-1) | 71.48±0.28b | 53.99±0.96c | 79.53±0.95a |

**Supplementary Table 4.** Detailed data for Figure S3.

|  | pH | SWC (%) | NH4+-N (mg kg-1) | NO3--N (mg kg-1) | TN (%) | TP (g kg-1) | TK (%) | SOC (%) | AK (g kg-1) | S-SC (μg d-1 g-1) | S-CL (μg d-1 g-1) | SL (μg d-1 g-1) | S-UE (μg d-1 g-1) | FDA (μg d-1 g-1) | SJ (μg d-1 g-1) |
| --- | --- | --- | --- | --- | --- | --- | --- | --- | --- | --- | --- | --- | --- | --- | --- |
| IN | 7.88 | 13.39 | 2.50 | 11.28 | 0.57 | 1.05 | 2.96 | 5.96 | 0.37 | 132.02 | 1895.46 | 13.29 | 2098.84 | 423.96 | 70.96 |
|  | 7.84 | 13.38 | 2.26 | 10.68 | 0.62 | 0.97 | 2.83 | 5.83 | 0.37 | 126.93 | 1747.69 | 12.62 | 2120.21 | 477.68 | 71.54 |
|  | 7.75 | 13.41 | 2.38 | 10.74 | 0.58 | 1.05 | 2.86 | 5.87 | 0.36 | 127.76 | 1734.46 | 14.30 | 2039.04 | 436.31 | 71.93 |
| ON | 7.39 | 12.77 | 2.74 | 15.17 | 0.60 | 0.99 | 2.96 | 5.53 | 0.34 | 120.98 | 2790.62 | 69.66 | 2034.11 | 411.02 | 52.22 |
|  | 7.40 | 12.13 | 2.34 | 14.80 | 0.59 | 0.99 | 2.95 | 5.42 | 0.35 | 129.85 | 2906.44 | 71.96 | 2102.56 | 452.93 | 55.52 |
|  | 7.47 | 12.57 | 2.51 | 15.01 | 0.61 | 0.99 | 2.96 | 5.55 | 0.34 | 124.38 | 2738.56 | 70.05 | 2102.92 | 467.55 | 54.24 |
| OUT | 7.69 | 13.25 | 1.75 | 11.52 | 0.56 | 1.02 | 3.04 | 5.32 | 0.38 | 120.50 | 3891.58 | 64.28 | 2296.83 | 535.81 | 77.77 |
|  | 7.60 | 12.13 | 2.19 | 11.63 | 0.56 | 0.99 | 3.09 | 5.34 | 0.38 | 123.68 | 4006.62 | 65.20 | 2365.64 | 538.30 | 79.77 |
|  | 7.62 | 12.87 | 2.10 | 11.66 | 0.56 | 1.00 | 3.08 | 5.34 | 0.38 | 123.72 | 3780.60 | 62.93 | 2270.13 | 545.28 | 81.04 |
